# Supplementary material for: Modulation of Long-Range Connectivity Patterns via Frequency-Specific Stimulation of Human Cortex
Source: Curr Biol. 2017 Oct 9;27(19):3061–3068.e3. doi: 10.1016/j.cub.2017.08.075 (PMC5640151; doi:10.1016/j.cub.2017.08.075)
Supplement: Document S1. Figures S1 and S2 [file mmc1.pdf]

Current Biology, Volume *27*

Supplemental Information

Modulation of Long-Range Connectivity Patterns via Frequency-Specific Stimulation of Human Cortex

Christiane A. Weinrich, John-Stuart Brittain, Magdalena Nowak, Reza Salimi-Khorshidi, Peter Brown, and Charlotte J. Stagg

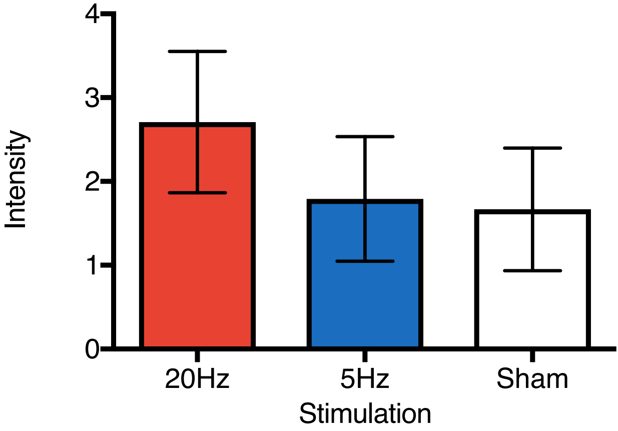
Intensity
4
3
2
1
0
20Hz
5Hz
Sham
Stimulation

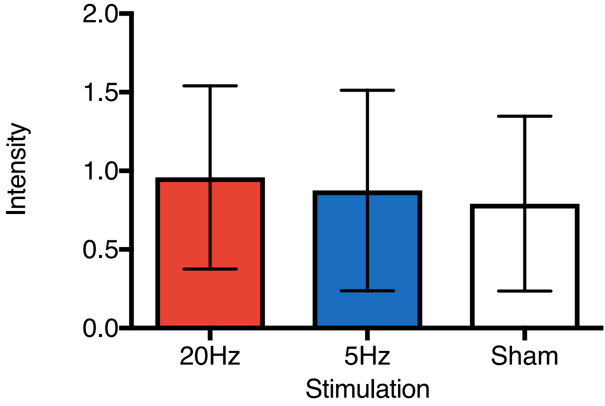
Intensity
2.0
1.5
1.0
0.5
0.0
20Hz
5Hz
Sham
Stimulation

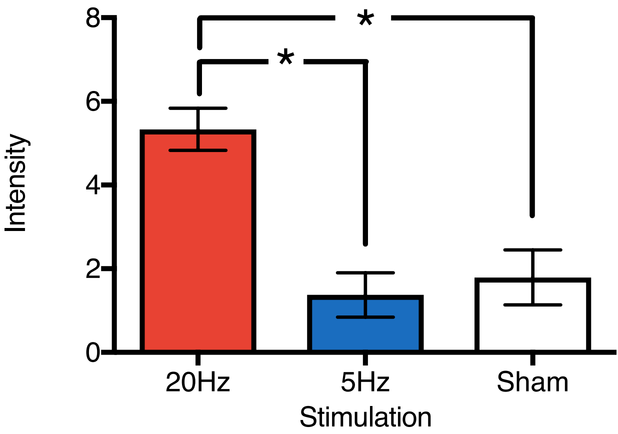
Intensity
8
6
4
2
0
*
*
20Hz
5Hz
Sham
Stimulation

Figure S1. Subject reported experience of tACS-induced phenomena, Related to Figure 1.

Paraesthesia (A), pain (B), and phosphenes (C) were rated on a visual analogue scale from 0 -10. There was no difference in the perception of paraesthesia and pain between the three stimulation conditions (20Hz, 5Hz and Sham) (Repeated Measures ANOVA: sensation: $F(2,22) = 1.73$, $p = 0.20$; pain: $F(1.108,12.186) = 0.33$ $p = 0.60$). However, there was a significant difference in the strength of phosphenes perceived between the three stimulation conditions (Repeated Measures ANOVA: $F(1.37, 15.01) = 28.52$, $p < 0.001$). Phosphenes perceived during tACS with this electrode montage are likely to be due to direct stimulation of the retina, rather than reflecting stimulation of the occipital cortex [S1]. Subsequent post hoc t-tests revealed 20Hz stimulation elicited a significantly stronger perception of phosphenes compared with both sham and 5Hz stimulation (20Hz versus sham: $t(11)=5.11$, $p < 0,001$; 20Hz versus 5Hz:, $t(11) = 6.20$, $p < 0.001$ [corrected]). There was no significant relationship between phosphene rating during 20Hz tACS and the strength of the motor network in response to 20Hz tACS ($r=0.32$, $p=0.30$), nor between phosphene rating and the strength of the DMN during 20Hz tACS (0.47, $p=0.11$).

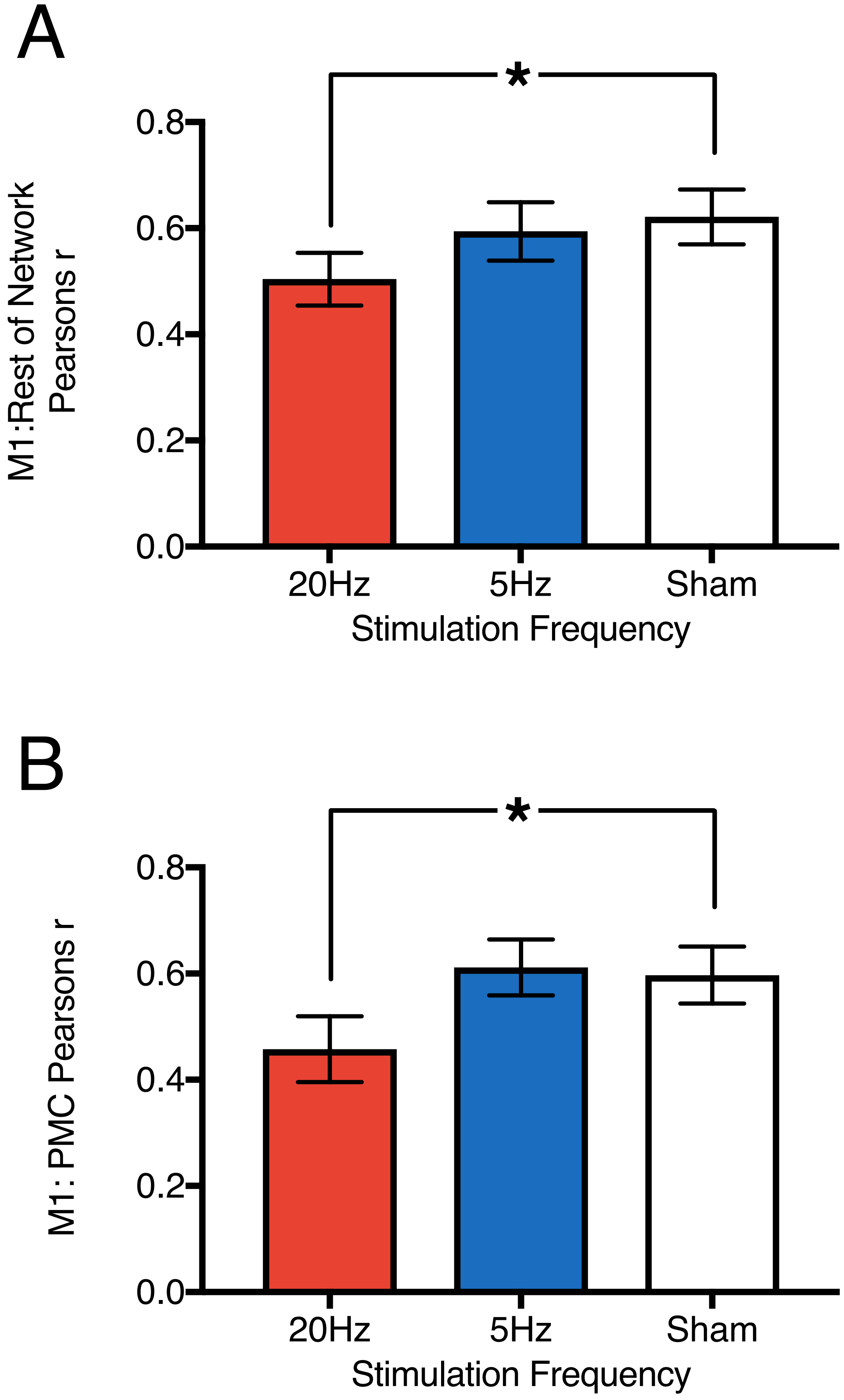

A
*
0.8
0.6
0.4
0.2
0.0
M1:Rest of Network
Pearsons r
20Hz
5Hz
Sham
Stimulation Frequency
B
*
0.8
0.6
0.4
0.2
0.0
M1: PMC Pearsons r
20Hz
5Hz
Sham
Stimulation Frequency

Figure S2. Region of Interest analyses. Related to Figure 2.

ROI analyses highlighting the change in connectivity between left M1 and (A) the motor network excluding the M1s bilaterally and (B) the left Premotor Cortex. Mean $\pm$ SEM, * $p < 0.05$.

Supplemental References

[S1] Schutter DJL, Hortensius R. (2010) Retinal origin of phosphenes to transcranial alternating current stimulation. Clin Neurophysiol 121, 1080–1084.
