# Supplementary material for: Modulation of Long-Range Connectivity Patterns via Frequency-Specific Stimulation of Human Cortex
Source: Curr Biol. 2017 Oct 9;27(19):3061–3068.e3. doi: 10.1016/j.cub.2017.08.075 (PMC5640151; doi:10.1016/j.cub.2017.08.075)
Supplement: Document S2. Article plus Supplemental Information [file mmc2.pdf]

Report

Current Biology

Modulation of Long-Range Connectivity Patterns via Frequency-Specific Stimulation of Human Cortex

Highlights

- tACS does not alter overall functional connectivity between major network nodes
- However, tACS modulates the connectivity pattern of the stimulated motor cortex
- These data directly support the "communication through coherence" hypothesis
- We provide evidence for how disordered connectivity arises from oscillatory changes

Authors

Christiane A. Weinrich, John-Stuart Brittain, Magdalena Nowak, Reza Salimi-Khorshidi, Peter Brown, Charlotte J. Stagg

Correspondence

charlotte.stagg@ndcn.ox.ac.uk

In Brief

Weinrich et al. show that entraining the phase of oscillations in one brain region modulates the connectivity pattern of that region without altering the strength of its connectivity with any specific region. These data provide evidence for how disordered functional communication arises in conditions characterized by perturbation of oscillations.

Weinrich et al., 2017, Current Biology *27*, 3061–3068
October 9, 2017 © 2017 The Authors. Published by Elsevier Ltd.
http://dx.doi.org/10.1016/j.cub.2017.08.075

CellPress

Current Biology
Report

Modulation of Long-Range Connectivity Patterns via Frequency-Specific Stimulation of Human Cortex

Christiane A. Weinrich,[1,2] John-Stuart Brittain,[3] Magdalena Nowak,[1,4] Reza Salimi-Khorshidi,[1,5] Peter Brown,[3,6] and Charlotte J. Stagg[1,4,6,7,8,*]
[1]Wellcome Centre for Integrative Neuroimaging, FMRIB, Nuffield Department of Clinical Neurosciences, University of Oxford, Oxford OX3 9DU, UK
[2]Department of Cognitive Neurology and Department of Neurology, University Medical Centre, University of Goettingen, Goettingen 37075, Germany
[3]Medical Research Council Brain Network Dynamics Unit and Nuffield Department of Clinical Neurosciences, University of Oxford, Oxford OX3 9DU, UK
[4]Oxford Centre for Human Brain Activity, Wellcome Centre for Integrative Neuroimaging, Department of Psychiatry, University of Oxford, Oxford OX3 7JX, UK
[5]George Institute for Global Health, Oxford Martin School, University of Oxford, Oxford OX1 3BD, UK
[6]These authors contributed equally
[7]Twitter: @cjstagg
[8]Lead Contact
*Correspondence: charlotte.stagg@ndcn.ox.ac.uk
http://dx.doi.org/10.1016/j.cub.2017.08.075

SUMMARY

There is increasing interest in how the phase of local oscillatory activity within a brain area determines the long-range functional connectivity of that area. For example, increasing convergent evidence from a range of methodologies suggests that beta (20 Hz) oscillations may play a vital role in the function of the motor system [1–5]. The "communication through coherence" hypothesis posits that the precise phase of coherent oscillations in network nodes is a determinant of successful communication between them [6, 7]. Here we set out to determine whether oscillatory activity in the beta band serves to support this theory within the cortical motor network in vivo. We combined non-invasive transcranial alternating-current stimulation (tACS) [8–12] with resting-state functional MRI (fMRI) [13] to follow both changes in local activity and long-range connectivity, determined by inter-areal blood-oxygen-level-dependent (BOLD) signal correlation, as a proxy for communication in the human cortex. Twelve healthy subjects participated in three fMRI scans with 20 Hz, 5 Hz, or sham tACS applied separately on each scan. Transcranial magnetic stimulation (TMS) at beta frequency has previously been shown to increase local activity in the beta band [14] and to modulate long-range connectivity within the default mode network [15]. We demonstrated that beta-frequency tACS significantly changed the connectivity pattern of the stimulated primary motor cortex (M1), without changing overall local activity or network connectivity. This finding is supported by a simple phase-precession model, which demonstrates the plausibility of the results and provides emergent predictions that are consistent with our empirical findings. These findings therefore inform our understanding of how local oscillatory activity may underpin network connectivity.

RESULTS

20 Hz tACS Does Not Alter Resting Activity in the Stimulated M1

We first wished to investigate whether 20 Hz transcranial alternating-current stimulation (tACS) would induce changes in cortical activity (Figure 1). A whole-brain voxel-wise analysis indicated that, as predicted, there was no net change in activity during 20 Hz stimulation compared with either 5 Hz tACS or sham tACS (thresholded at $Z > 2.3$, $p = 0.05$ (corrected)). Additionally, there were no activity changes during 20 Hz tACS within our pre-specified regions of interest (ROIs; left primary motor cortex [M1], right M1, left premotor cortex [PMC], and right PMC; see STAR Methods for details; repeated-measures ANOVA [RM-ANOVA]: no main effect of ROI [$F(2,22) = 3.093$, $p = 0.071$], no main effect of tACS condition [$F(2,22) = 1.920$, $p = 0.17$], and no interaction between ROI and stimulus condition [$F(6, 66) = 0.907$ $p = 0.495$]).

20 Hz tACS Does Not Modulate Overall Motor Network Connectivity

We primarily wished to investigate the effects of M1 tACS on functional connectivity across the motor network. To assess the strength of connections between key network nodes, we used both ROI seed-based analyses and an independent-component analysis (ICA) approach, which identifies spatially distinct networks of regions with correlated resting blood-oxygen-level-dependent (BOLD) activity, the so-called resting-state networks (RSNs) [16–18]. For the ICA analyses, we were primarily interested in the motor network and additionally

© 2017 The Authors. Published by Elsevier Ltd.
This is an open access article under the CC BY license (http://creativecommons.org/licenses/by/4.0/).

CellPress

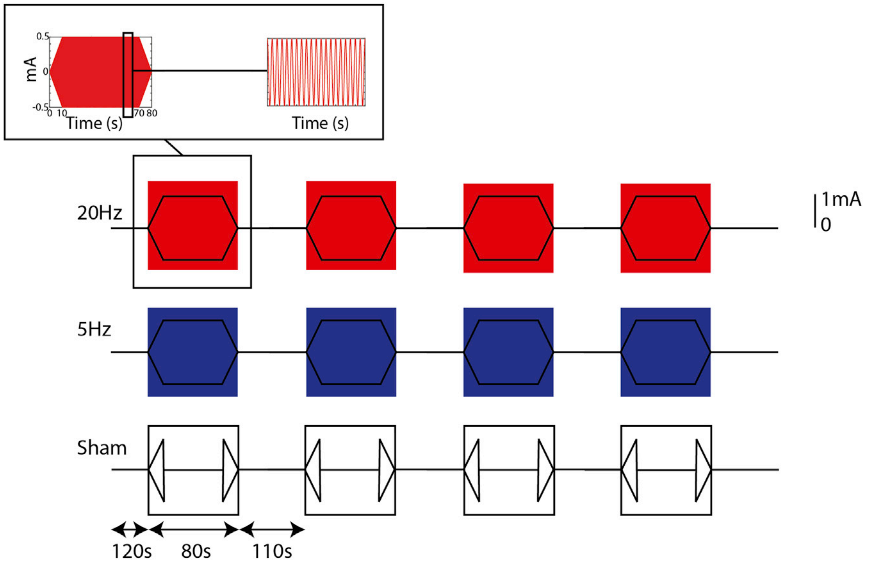

Figure 1. Experimental Outline

Each participant had three scans, acquired on the same day, and during which 20 Hz, 5 Hz, or sham tACS was applied with a sinusoidal waveform and no current offset to the left M1, with the order counterbalanced across the group. Real tACS was performed for 60 s, with 10 s ramp-up and ramp-down periods on either side, repeated four times in each run. Sham stimulation consisted of 10 s ramp up and ramp down only. There were 110 s between each stimulation period. Subjects were advised to keep their eyes open and to look at a cross displayed centrally throughout the scans. Insets show details of the current amplitude for each stimulation period. Participants were asked to rate levels of paraesthesia, pain, and phosphenes on visual analog scales between each scan. See also Figure S1.

identified the default mode network (DMN), a well characterized network that does not include M1, as an anatomical control. Network strength gives a measure of functional connectivity within that network and has been shown to be a sensitive metric for better communication between the major network nodes [19, 20].

We first performed an ROI analysis to investigate changes in functional connectivity during tACS between the stimulated (left) M1 and other major network nodes (right M1; left PMC). There were no significant changes in M1 connectivity with 20 Hz tACS compared with the other frequencies (RM-ANOVA, one factor of frequency [20 Hz, 5 Hz, sham] and one factor of ROI [right M1, left PMC]: main effect of frequency, $F(2,22)$ = 0.87, $p = 0.431$; frequency × ROI interaction, $F(2,22)$ = 8.13, $p = 0.002$; M1-M1 connectivity shown in Figure 2A). We went on to explore the frequency by ROI interaction and demonstrated no frequency-specific changes in M1-M1 connectivity (RM-ANOVA, one factor of frequency [20 Hz, 5 Hz, Sham], $F(2,22)$ = 2.30, $p = 0.13$) but a significant decrease in M1-PMC connectivity during 20 Hz tACS (RM-ANOVA, $F(2,22)$ = 9.51, $p = 0.001$; post hoc t test [20 Hz v Sham], $t(11) = 4.1$, $p = 0.002$; Figure S2A).

We next investigated tACS-related changes in connectivity within subject-specific motor network and DMN maps (Figure 3; see STAR Methods for details of subject-specific networks derivation [21]). There were no frequency-specific changes in network strength in the motor network or the DMN (RM-ANOVA, one factor of frequency (20 Hz, 5 Hz, sham), one factor of network (motor, DMN); main effect of frequency, $F(2,22)$ = 2.902, $p = 0.76$; frequency × network interaction, $F(2,22)$ = 2.083, $p = 0.148$; Figures 2B and 2C).

20 Hz tACS Uncouples Components of the Motor Network

It has previously been demonstrated that M1-M1 connectivity and motor RSN strength are ordinarily closely related [21]. Our primary hypothesis was that stimulation of the left M1 at its resting resonant frequency would entrain left M1, together with its strongest reciprocal connections, to the extrinsic rhythm. The inter-areal connectivity, as judged by functional MRI (fMRI), would not necessarily be expected to change, but the phase of activity would be drawn away from the steady-state phase relationships established in the remainder of the motor RSN. Therefore, the strong inter-areal connectivity between the M1s would be uncoupled from the remainder of the motor RSN.

As expected, there was a strong correlation between M1-M1 connectivity and motor network strength in the sham condition (R^2 = 0.74, $p < 0.001$; Figure 2F). However, this relationship was lost during 20 Hz stimulation (R^2 = 0.05, $p = 0.47$; 20 Hz versus sham, Fisher's r-to-Z: $Z = 2.25$, $p = 0.024$; Figure 2D). Critically, this uncoupling was frequency selective and not observed with 5 Hz tACS (5 Hz: R^2 = 0.74, $p < 0.001$; 20 Hz versus 5 Hz, Fisher's r-to-Z: $Z = 2.23$, $p = 0.026$; Figure 2E). No significant correlation was observed between M1-M1 connectivity and DMN strength at any tACS frequency (Figures 2G–2I).

To confirm an uncoupling between left M1 and the rest of the motor network, we additionally performed an ROI analysis to investigate connectivity between the left M1 and the motor network excluding both M1s (rest of network). This confirmed a frequency-specific decrease in connectivity between left M1 and the rest of the network during 20 Hz stimulation compared with sham (Figure S2B, RM-ANOVA, main effect of stimulation: $F(2,22)$ = 4.243, $p = 0.02$; follow-up t test between 20 Hz and sham: $t(11) = 3.168$, $p = 0.009$).

However, entraining M1 oscillations with 20 Hz tACS might be expected to act as noise in circuits that are not strongly connected to M1 and may not mirror its resonance profile. We therefore calculated the strength of the connectivity in areas within the motor network but outside the M1s. There was a significant decrease in connectivity in these areas with 20 Hz compared to sham ($t(11) = 2.31$, $p = 0.04$), supporting the hypothesis that the tACS-entrained oscillations in M1 were insufficient to fully capture phase relationships within secondary motor areas but still managed to weaken existing phase alignments favoring communication.

CellPress

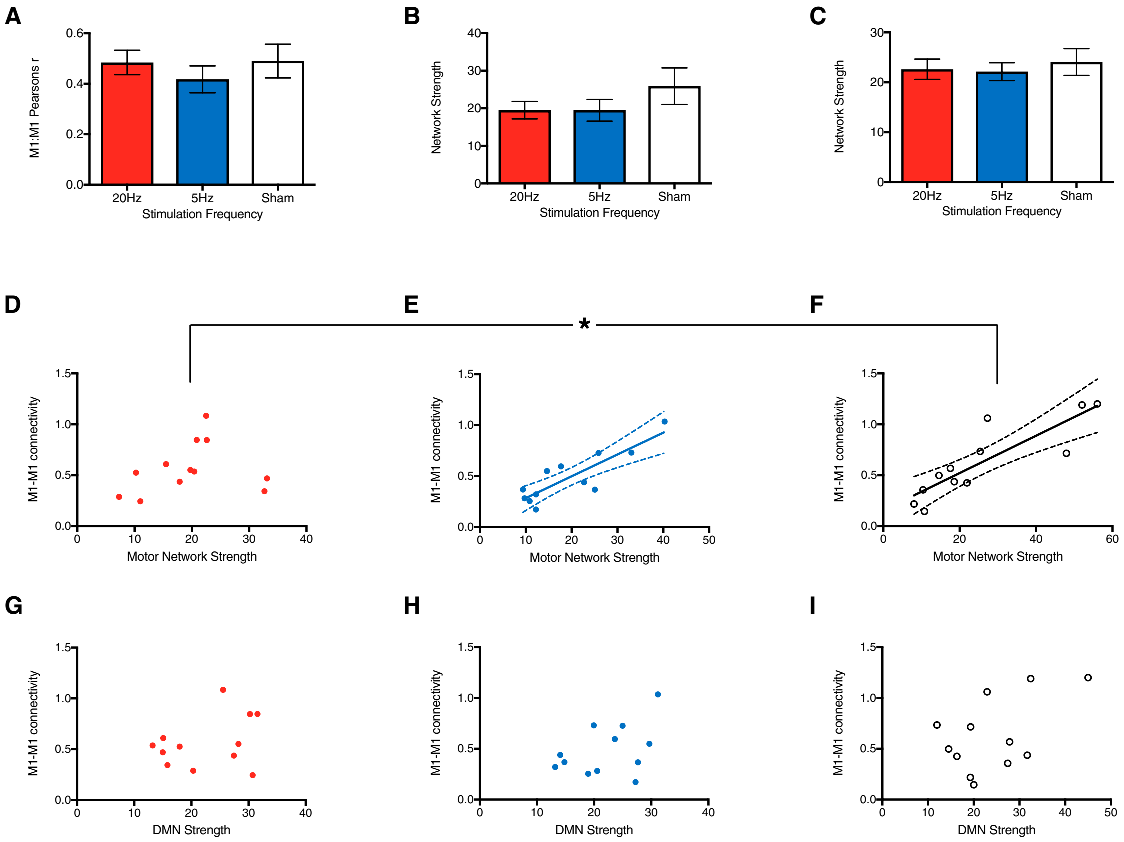

Figure 2. Modulation of the Relationship between M1-M1 Connectivity and Motor Network Connectivity

(A) ROI analysis showed 20 Hz stimulation did not change M1-M1 connectivity compared to either 5 Hz or sham stimulation. Bars indicate mean ± SEM.

(B and C) Similarly, there was no change in overall network strength in either the motor network (B) or default mode network (DMN) (C) as a result of stimulation. Bars indicate mean ± SEM.

(D–F) Relationship between motor network strength and M1-M1 connectivity. The expected close relationship between M1-M1 connectivity and motor network strength, seen with both sham (F) and 5 Hz (E) stimulation was lost with 20 Hz stimulation (D), suggesting that the pattern of connectivity within the motor network was significantly changed by local stimulation at the beta frequency. Results of linear regression and 95% confidence limits shown in (E) and (F).

(G–I) As expected, the relationship between M1-M1 connectivity and RSN strength was anatomically specific, with no correlation between M1-M1 connectivity and DMN strength. As Pearson's correlation coefficient is not normally distributed, an r-to-Z transformation was performed for all measures of M1-M1 connectivity. Network strength is calculated as the mean parameter estimate across the network and is given in arbitrary units.

The asterisk indicates significant difference ($p < 0.05$) between sham stimulation and 20 Hz stimulation in the relationship between M1-M1 connectivity and motor network strength. See also Figure S2.

Finally, we wished to investigate whether there were any systematic differences in signal to noise ratio (SNR) across our three scan sessions that might explain our results. We therefore calculated the SNR in each scan session for each subject separately and demonstrated no significant differences in SNR between any of the three stimulation conditions (thresholded at $Z > 2.3$, $p = 0.05$ [corrected]).

A Simple Phase-Precession Model Explains Key Features of Our Data

Taken together, our findings point toward a system of coupled brain regions that rely on temporal channels of synchronous bursting for communication, and that moderately weak stimulation (tACS) close to the resonant frequency of this system acts to actively decouple the constituent elements of the network. We wanted to demonstrate the feasibility of such a scenario by constructing a mathematical model that relies only on the principles of phase precession (Figure 4A).

This model was able to faithfully reproduce key features of our experimental data. These included: (1) the frequency-specific loss of association between M1-M1 and overall motor network strength ($R^2 = 0.913$ without stimulation versus $R^2 = 0.130$ during 20 Hz stimulation; difference of correlation test, $z = -10.5$, $p < 0.001$; 5 Hz stimulation had no discernible effect, $R^2 = 0.902$, $z = -0.430$, $p = 0.667$); (2) a strong reduction in net coupling of the secondary motor system (Cohen's $d = -5.183$), absent with 5 Hz stimulation; and (3) relatively preserved M1-M1 coupling (Cohen's $d = 1.391$; Figures 4B–4D).

Our model additionally predicted that a M1-M1 loop with intrinsically weak connectivity is more readily entrained by 20 Hz (but not 5 Hz) tACS than a more synchronized loop, which may even begin to desynchronize (Figure 4E). This was found to

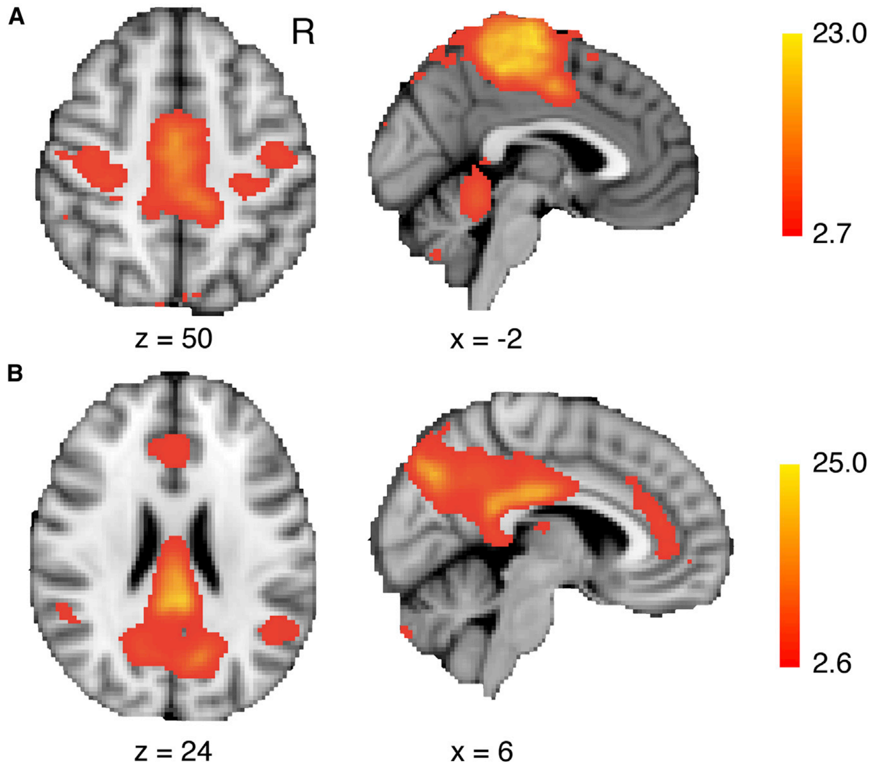

Figure 3. Resting State Networks

ICA-derived group mean (A) motor resting state network and (B) default mode network.

be the case in a secondary analysis of the fMRI data, where we related M1-M1 connectivity during sham stimulation, as a baseline metric, to the change in M1-M1 connectivity between 20 Hz tACS and sham stimulation ($R^2 = 0.52$, $p = 0.008$; Figure 4F). Note that while this model captures (even predicts) many of the characteristics observed in our experimental data, it represents a single topology tuned independently of the data itself and employs phase synchronization as a proxy for correlations in BOLD activity. As such, the dynamic range of the model will be far narrower than that expected from the experimental data, which is considered across subjects.

DISCUSSION

This study was performed to investigate the role of M1 beta oscillations in determining the network-level functional connectivity of the region. tACS is believed to superimpose alternating subthreshold windows of excitation and inhibition on underlying neurons, with this effect being more pronounced through local resonance when stimulation is delivered at the relevant frequencies [22]. Accordingly, we delivered tACS at 20 Hz over the M1 at rest with the intention of entraining local beta activity, thereby shifting the phase of beta oscillations away from that dictated by the steady-state dynamics of the wider motor network. Although weak currents would not be predicted to change the overall level of local activity, they might be proposed to draw M1 and its strongest reciprocal connections away from those phase relationships with the remainder of the motor network that would otherwise be optimal for communication [23]. This effect might by induced by synchronization of the neural firing to the extra-cranially applied sinusoidal current as has been shown in a rat model [24], and increases in beta activity have been demonstrated after 20 Hz rTMS [14].

We tested 20 Hz, 5 Hz, and sham tACS applied to the left M1 during resting state fMRI and found that while beta-frequency tACS led to no significant overall change in M1-M1 connectivity or in motor network strength, it changed the relationship between these two metrics. Previous work has established a significant relationship between connectivity between the left and right M1 and connectivity within the motor network as a whole [21, 25], reflecting the role of the M1s as the major nodes within this network. Although the study was limited to 12 participants and therefore might not have detected subtle changes in connectivity, the finding that beta-frequency tACS significantly modulates this relationship is important, as it suggests that changing the phase of local activity at the resonant frequency of M1 can modulate the connectivity pattern of that area. It should be noted that a direct effect of the reference electrode on the supra-orbital ridge cannot be ruled out. Similarly, it is possible that some of the current from the electrode positioned over M1 spread to surrounding cortical regions, such as the premotor cortices. However, the M1-specific findings demonstrated here are consistent with the major effects of stimulation being maximal over this site, perhaps because the 20 Hz stimulation frequency was deliberately chosen to be close to the local resonance frequency.

A parsimonious explanation for the results seen here might be that the substantial structural connections between the two M1s and their similar resonance characteristics means that the phase of both left and right M1s becomes relatively entrained by 20 Hz tACS, leading to greater maintenance of phase coupling and therefore correlated fluctuations in BOLD signal between these primary motor areas. However, the capture of phase by extrinsic tACS uncouples M1-M1 connectivity from the remainder of the motor network so that the fluctuations in BOLD signal between the two subcomponents are no longer correlated. In practice, though, it is likely that MI activity also influences phase relationships, and hence communication, between non-primary motor regions. Hence, on closer inspection, when connectivity within secondary motor areas was considered alone, uncoupling of M1 from the remainder of the motor network did decrease network connectivity in other motor regions. Presumably, in this case, connections with M1 are weaker and resonance characteristics less well matched, so that M1 entrainment acts as a source of noise, partially disrupting those optimal phase relationships for communication that may spontaneously emerge within

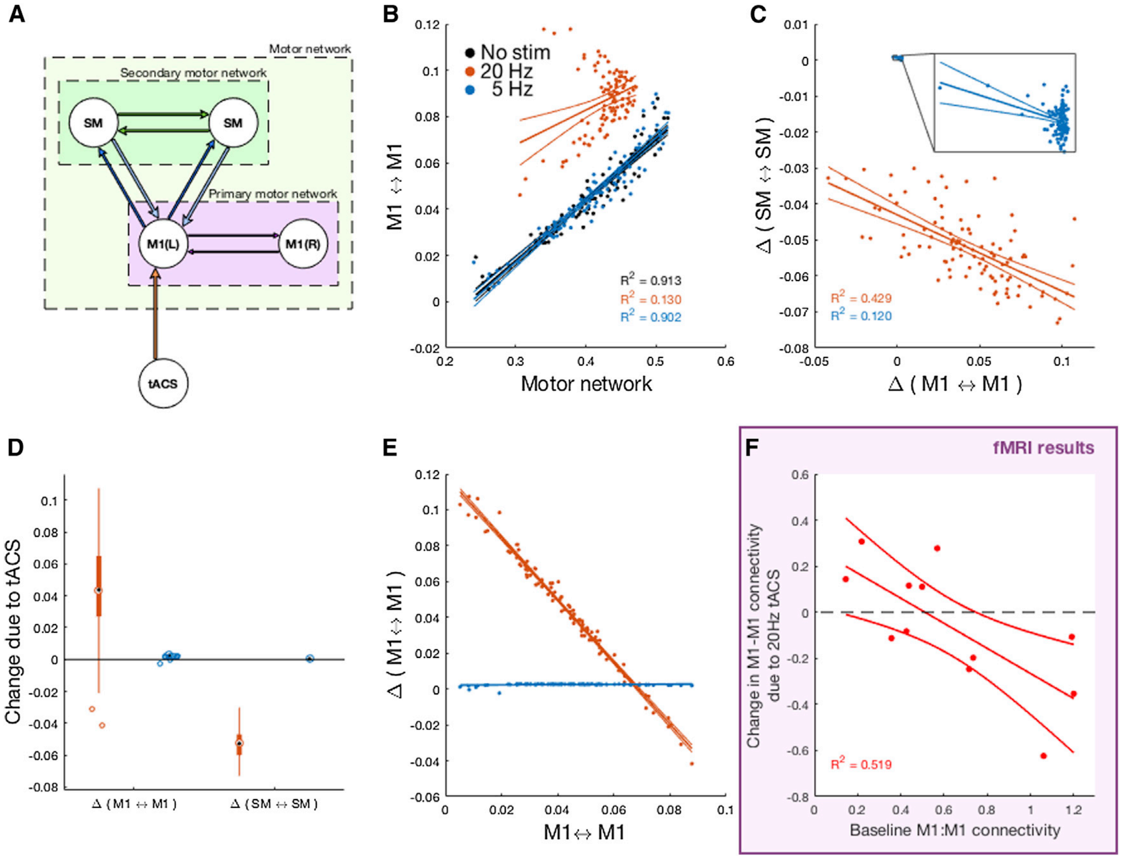

Figure 4. Phase-Precession Model

(A) Model topology.

(B–F) The model was able to faithfully reproduce key features of the observed experimental data, including (B) the (frequency-specific) loss of association between M1-M1 and overall motor network strength and (C and D) the reduction in net coupling of the secondary motor system, despite relatively preserved M1-M1 coupling. Our model additionally predicted that a M1-M1 loop with intrinsically weak connectivity is more readily entrained by 20 Hz (but not 5 Hz) tACS, whereas a more synchronized loop is less affected and may even begin to desynchronize (E). Baseline M1-M1 connectivity is plotted on the x axis, change in M1-M1 connectivity due to 20 Hz tACS on the y axis. This was confirmed by a secondary analysis of the fMRI data (F). Δ, change in PSI due to stimulation.

secondary motor areas [26]. The finding that 20 Hz tACS decreases M1-PMd connectivity in a manner that significantly co-varied with the effects of 20 Hz tACS on M1-M1 connectivity further supports the conclusion that tACS at 20 Hz entrained the phase of underlying M1 oscillations, which in turn affects the phase relationships between connected regions. In order to explore the plausibility of this hypothesis, we developed a simple-phase-precession model, which was able to explain key aspects of our data, as well as provide emergent properties that we were able to support with our empirical data.

It is important to note that the functional connectivity modulations were quick to emerge and observed during relatively short periods of tACS. We specifically designed this experiment to avoid tACS aftereffects as we wished to focus on entrainment effects during stimulation, and we use repeated stimulation periods within the same scanning period to reduce across-session variation in BOLD fluctuations. Convincing tACS-related behavioral effects have previously been observed during short-periods of tACS [9, 11], leading to the speculation that these effects may be underpinned, at least in part, by the functional connectivity changes observed here.

Communication through Coherence Hypothesis

We directly tested a commonly held theory that proposes that the efficacy of information flow between different brain areas depends on the phase alignment between their intrinsic activities, the so-called "communication through coherence" hypothesis. According to this hypothesis, optimal phase alignment between functionally connected cortical areas predicts an increase in correlation between the two areas, where correlation is a proxy for information exchange [6, 7, 27]. In practice, such optimal phase alignment may be the natural result of the steady-state dynamics of interconnected neural circuits [26]. Our findings, while necessarily indirect to avoid confounds that can occur with electroencephalographic (EEG) and magnetoencephalographic (MEG) signals due to electrical stimulation [12, 25], nevertheless provide empirical support for the "communication through coherence" hypothesis. Further, our data suggest that the impact of phase capture of one area upon phase relationships in interconnected regions may be dependent on the strength of recurrent connectivity and resonance characteristics. It will be important for future studies to confirm these findings with EEG or MEG approaches and to determine the extent to which task-dependent

changes in recurrent connectivity and local resonances modify entrainment.

Other Applicable Hypotheses

The data presented here are also open to interpretation in the context of other theories of inter-areal communication. For example, it has been proposed that power-power coupling between areas is crucial for communication [26]. In that MEG study power envelope correlations occurred on the timescale of several seconds. These therefore overlap with the frequencies of BOLD covariation detected in the present study, raising the intriguing possibility that the previously reported power-power coupling and the present findings may be related. Indeed, if one assumes that phase synchronization across neural populations leads to increased post-synaptic efficacy through temporal integration and thereby improved communication, then low-frequency dynamics in phase synchronization should be translated in to low-frequency amplitude covariations. Whether phase synchronization (coherence) or power coupling are the mechanistically important aspect supporting neural communication then becomes an issue for empirical testing [27].

The Role of Beta Oscillations

The beta band frequency forms the dominant oscillatory activity in the primary motor cortex at rest [3, 4]. In general, inter-areal connectivity, quantified here via resting-state fMRI, is thought to be primarily driven by alpha and beta oscillatory activity within key network nodes [28–32], evidence supported by a recent study demonstrating that alpha-frequency tACS led to significant modulation of functional connectivity [33], though necessarily this evidence is indirect.

However, this does not exclude a role for activity at other frequencies, particularly the gamma band, in network-level connectivity [28, 34]. Indeed, it might be that stimulating at the gamma frequency would lead to a change in the overall connectivity within the network, especially during movement, and this should be investigated directly in future studies.

This study is, to our knowledge, the first to explore the role of modulating M1 beta phase in network-level connectivity and lends substantial support to the hypothesis that phase coupling between anatomically distant regions underpins functional connectivity and therefore communication, between them.

STAR★METHODS

Detailed methods are provided in the online version of this paper and include the following:

- KEY RESOURCES TABLE
- CONTACT FOR REAGENT AND RESOURCE SHARING
- EXPERIMENTAL MODEL AND SUBJECT DETAILS
- METHOD DETAILS
  - MR acquisition
  - Transcranial alternating current stimulation (tACS)
  - Image analysis
  - Phase-precession model
- QUANTIFICATION AND STATISTICAL ANALYSIS
  - Whole Brain Analyses
  - Region of Interest Analyses

SUPPLEMENTAL INFORMATION

Supplemental Information includes two figures and can be found with this article online at http://dx.doi.org/10.1016/j.cub.2017.08.075.

AUTHOR CONTRIBUTIONS

Conceptualization, C.W., P.B., and C.J.S.; Methodology, J.-S.B.; Investigation, C.W. and M.N.; Formal Analysis, R.S.-K.; Writing – Original Draft, C.W., P.B., and C.J.S.; Writing – Review & Editing, C.W., J.-S.B., M.N., R.S.-K., P.B., and C.J.S.; Funding Acquisition, P.B.; Supervision, P.B. and C.J.S.

ACKNOWLEDGMENTS

M.N. is funded by the Wellcome Trust. J.S.B. is funded by the Medical Research Council (MR/N003446/1). P.B. is funded by the Medical Research Council (MC_UU_12024/1) and the National Institute of Health Research (NIHR) Oxford Biomedical Research Centre. C.J.S. holds a Sir Henry Dale Fellowship, funded by the Wellcome Trust and the Royal Society (102584/Z/13/Z). The work was supported by the NIHR Oxford Health Biomedical Research Centre. The Wellcome Centre for Integrative Neuroimaging is supported by core funding from the Wellcome Trust (203139/Z/16/Z).

Received: February 8, 2017
Revised: July 26, 2017
Accepted: August 30, 2017
Published: September 28, 2017

REFERENCES

1. Salmelin, R., and Hari, R. (1994). Characterization of spontaneous MEG rhythms in healthy adults. Electroencephalogr. Clin. Neurophysiol. *91*, 237–248.
2. Gilbertson, T., Lalo, E., Doyle, L., Di Lazzaro, V., Cioni, B., and Brown, P. (2005). Existing motor state is favored at the expense of new movement during 13-35 Hz oscillatory synchrony in the human corticospinal system. J. Neurosci. *25*, 7771–7779.
3. Baker, S.N., Olivier, E., and Lemon, R.N. (1997). Coherent oscillations in monkey motor cortex and hand muscle EMG show task-dependent modulation. J. Physiol. *501*, 225–241.
4. Pfurtscheller, G., and Lopes da Silva, F.H. (1999). Event-related EEG/MEG synchronization and desynchronization: basic principles. Clin. Neurophysiol. *110*, 1842–1857.
5. Brittain, J.-S., Sharott, A., and Brown, P. (2014). The highs and lows of beta activity in cortico-basal ganglia loops. Eur. J. Neurosci. *39*, 1951–1959.
6. Fries, P. (2005). A mechanism for cognitive dynamics: neuronal communication through neuronal coherence. Trends Cogn. Sci. *9*, 474–480.
7. Fries, P. (2009). Neuronal gamma-band synchronization as a fundamental process in cortical computation. Annu. Rev. Neurosci. *32*, 209–224.
8. Thut, G., Miniussi, C., and Gross, J. (2012). The functional importance of rhythmic activity in the brain. Curr. Biol. *22*, R658–R663.
9. Pogosyan, A., Gaynor, L.D., Eusebio, A., and Brown, P. (2009). Boosting cortical activity at Beta-band frequencies slows movement in humans. Curr. Biol. *19*, 1637–1641.
10. Kanai, R., Paulus, W., and Walsh, V. (2010). Transcranial alternating current stimulation (tACS) modulates cortical excitability as assessed by TMS-induced phosphene thresholds. Clin. Neurophysiol. *121*, 1551–1554.
11. Joundi, R.A., Jenkinson, N., Brittain, J.-S., Aziz, T.Z., and Brown, P. (2012). Driving oscillatory activity in the human cortex enhances motor performance. Curr. Biol. *22*, 403–407.
12. Helfrich, R.F., Schneider, T.R., Rach, S., Trautmann-Lengsfeld, S.A., Engel, A.K., and Herrmann, C.S. (2014). Entrainment of brain oscillations by transcranial alternating current stimulation. Curr. Biol. *24*, 333–339.

13. Cabral-Calderin, Y., Williams, K.A., Opitz, A., Dechent, P., and Wilke, M. (2016). Transcranial alternating current stimulation modulates spontaneous low frequency fluctuations as measured with fMRI. Neuroimage *141*, 88–107.

14. Romei, V., Bauer, M., Brooks, J.L., Economides, M., Penny, W., Thut, G., Driver, J., and Bestmann, S. (2016). Causal evidence that intrinsic beta-frequency is relevant for enhanced signal propagation in the motor system as shown through rhythmic TMS. Neuroimage *126*, 120–130.

15. Eldaief, M.C., Halko, M.A., Buckner, R.L., and Pascual-Leone, A. (2011). Transcranial magnetic stimulation modulates the brain's intrinsic activity in a frequency-dependent manner. Proc. Natl. Acad. Sci. USA *108*, 21229–21234.

16. Raichle, M.E., MacLeod, A.M., Snyder, A.Z., Powers, W.J., Gusnard, D.A., and Shulman, G.L. (2001). A default mode of brain function. Proc. Natl. Acad. Sci. USA *98*, 676–682.

17. Beckmann, C.F., DeLuca, M., Devlin, J.T., and Smith, S.M. (2005). Investigations into resting-state connectivity using independent component analysis. Philos. Trans. R. Soc. Lond. B Biol. Sci. *360*, 1001–1013.

18. Fox, M.D., and Raichle, M.E. (2007). Spontaneous fluctuations in brain activity observed with functional magnetic resonance imaging. Nat. Rev. Neurosci. *8*, 700–711.

19. Čeko, M., Gracely, J.L., Fitzcharles, M.A., Seminowicz, D.A., Schweinhardt, P., and Bushnell, M.C. (2015). Is a responsive default mode network required for successful working memory task performance? J. Neurosci. *35*, 11595–11605.

20. Tavor, I., Parker Jones, O., Mars, R.B., Smith, S.M., Behrens, T.E.J., and Jbabdi, S. (2016). Task-free MRI predicts individual differences in brain activity during task performance. Science *352*, 216–220.

21. Stagg, C.J., Bachtiar, V., Amadi, U., Gudberg, C.A., Ilie, A.S., Sampaio-Baptista, C., O'Shea, J., Woolrich, M., Smith, S.M., Filippini, N., et al. (2014). Local GABA concentration is related to network-level resting functional connectivity. eLife *3*, e01465.

22. Guerra, A., Pogosyan, A., Nowak, M., Tan, H., Ferreri, F., Di Lazzaro, V., and Brown, P. (2016). Phase dependency of the human primary motor cortex and cholinergic inhibition cancelation during beta tACS. Cereb. Cortex *26*, 3977–3990.

23. Cagnan, H., Duff, E.P., and Brown, P. (2015). The relative phases of basal ganglia activities dynamically shape effective connectivity in Parkinson's disease. Brain *138*, 1667–1678.

24. Ozen, S., Sirota, A., Belluscio, M.A., Anastassiou, C.A., Stark, E., Koch, C., and Buzsáki, G. (2010). Transcranial electric stimulation entrains cortical neuronal populations in rats. J. Neurosci. *30*, 11476–11485.

25. Bachtiar, V., Near, J., Johansen-Berg, H., and Stagg, C.J. (2015). Modulation of GABA and resting state functional connectivity by transcranial direct current stimulation. eLife *4*, e08789.

26. Hahn, G., Bujan, A.F., Frégnac, Y., Aertsen, A., and Kumar, A. (2014). Communication through resonance in spiking neuronal networks. PLoS Comput. Biol. *10*, e1003811.

27. Womelsdorf, T., Schoffelen, J.-M., Oostenveld, R., Singer, W., Desimone, R., Engel, A.K., and Fries, P. (2007). Modulation of neuronal interactions through neuronal synchronization. Science *316*, 1609–1612.

28. Hipp, J.F., Hawellek, D.J., Corbetta, M., Siegel, M., and Engel, A.K. (2012). Large-scale cortical correlation structure of spontaneous oscillatory activity. Nat. Neurosci. *15*, 884–890.

29. Lu, H., Zuo, Y., Gu, H., Waltz, J.A., Zhan, W., Scholl, C.A., Rea, W., Yang, Y., and Stein, E.A. (2007). Synchronized delta oscillations correlate with the resting-state functional MRI signal. Proc. Natl. Acad. Sci. USA *104*, 18265–18269.

30. Mantini, D., Perrucci, M.G., Del Gratta, C., Romani, G.L., and Corbetta, M. (2007). Electrophysiological signatures of resting state networks in the human brain. Proc. Natl. Acad. Sci. USA *104*, 13170–13175.

31. Brookes, M.J., Wood, J.R., Stevenson, C.M., Zumer, J.M., White, T.P., Liddle, P.F., and Morris, P.G. (2011). Changes in brain network activity during working memory tasks: a magnetoencephalography study. Neuroimage *55*, 1804–1815.

32. Wang, L., Saalmann, Y.B., Pinsk, M.A., Arcaro, M.J., and Kastner, S. (2012). Electrophysiological low-frequency coherence and cross-frequency coupling contribute to BOLD connectivity. Neuron *76*, 1010–1020.

33. Bächinger, M., Zerbi, V., Moisa, M., Polanía, R., Liu, Q., Mantini, D., Ruff, C., and Wenderoth, N. (2017). Concurrent tACS-fMRI reveals causal influence of power synchronized neural activity on resting state fMRI connectivity. J. Neurosci. *37*, 4766–4777.

34. Cabral, J., Hugues, E., Sporns, O., and Deco, G. (2011). Role of local network oscillations in resting-state functional connectivity. Neuroimage *57*, 130–139.

35. Oldfield, R.C. (1971). The assessment and analysis of handedness: the Edinburgh inventory. Neuropsychologia *9*, 97–113.

36. Moeller, S., Yacoub, E., Olman, C.A., Auerbach, E., Strupp, J., Harel, N., and Uğurbil, K. (2010). Multiband multislice GE-EPI at 7 tesla, with 16-fold acceleration using partial parallel imaging with application to high spatial and temporal whole-brain fMRI. Magn. Reson. Med. *63*, 1144–1153.

37. Xu, J., Moeller, S., Auerbach, E.J., Strupp, J., Smith, S.M., Feinberg, D.A., Yacoub, E., and Uğurbil, K. (2013). Evaluation of slice accelerations using multiband echo planar imaging at 3 T. Neuroimage *83*, 991–1001.

38. Smith, S.M., Jenkinson, M., Woolrich, M.W., Beckmann, C.F., Behrens, T.E., Johansen-Berg, H., Bannister, P.R., De Luca, M., Drobnjak, I., Flitney, D.E., et al. (2004). Advances in functional and structural MR image analysis and implementation as FSL. Neuroimage *23* (*Suppl. 1*), S208–S219.

39. Jenkinson, M., Beckmann, C.F., Behrens, T.E.J., Woolrich, M.W., and Smith, S.M. (2012). FSL. Neuroimage *62*, 782–790.

40. Woolrich, M.W., Jbabdi, S., Patenaude, B., Chappell, M., Makni, S., Behrens, T., Beckmann, C., Jenkinson, M., and Smith, S.M. (2009). Bayesian analysis of neuroimaging data in FSL. Neuroimage *25* (*Suppl. 1*), S173–S186.

41. Jenkinson, M., Bannister, P., Brady, M., and Smith, S. (2002). Improved optimization for the robust and accurate linear registration and motion correction of brain images. Neuroimage *17*, 825–841.

42. Kochiyama, T., Morita, T., Okada, T., Yonekura, Y., Matsumura, M., and Sadato, N. (2005). Removing the effects of task-related motion using independent-component analysis. Neuroimage *25*, 802–814.

43. Salimi-Khorshidi, G., Douaud, G., Beckmann, C.F., Glasser, M.F., Griffanti, L., and Smith, S.M. (2014). Automatic denoising of functional MRI data: combining independent component analysis and hierarchical fusion of classifiers. Neuroimage *90*, 449–468.

44. Power, J.D., Mitra, A., Laumann, T.O., Snyder, A.Z., Schlaggar, B.L., and Petersen, S.E. (2014). Methods to detect, characterize, and remove motion artifact in resting state fMRI. Neuroimage *84*, 320–341.

45. Stagg, C.J., O'Shea, J., Kincses, Z.T., Woolrich, M., Matthews, P.M., and Johansen-Berg, H. (2009). Modulation of movement-associated cortical activation by transcranial direct current stimulation. Eur. J. Neurosci. *30*, 1412–1423.

46. Tomassini, V., Jbabdi, S., Klein, J.C., Behrens, T.E., Pozzilli, C., Matthews, P.M., Rushworth, M.F., and Johansen-Berg, H. (2007). Diffusion-weighted imaging tractography-based parcellation of the human lateral premotor cortex identifies dorsal and ventral subregions with anatomical and functional specializations. J. Neurosci. *27*, 10259–10269.

47. Filippini, N., MacIntosh, B.J., Hough, M.G., Goodwin, G.M., Frisoni, G.B., Smith, S.M., Matthews, P.M., Beckmann, C.F., and Mackay, C.E. (2009). Distinct patterns of brain activity in young carriers of

the APOE-epsilon4 allele. Proc. Natl. Acad. Sci. USA *106*, 7209–7214.

48. Beckmann, C.F., Mackay, C.E., Filippini, N., and Smith, S.M. (2009). Group comparison of resting-state FMRI data using multi-subject ICA and dual regression. Neuroimage *47*, S148.

49. Cabral, J., Luckhoo, H., Woolrich, M., Joensson, M., Mohseni, H., Baker, A., Kringelbach, M.L., and Deco, G. (2014). Exploring mechanisms of spontaneous functional connectivity in MEG: how delayed network interactions lead to structured amplitude envelopes of band-pass filtered oscillations. Neuroimage *90*, 423–435.

50. Nowak, M., Hinson, E., van Ede, F., Pogosyan, A., Guerra, A., Quinn, A., Brown, P., and Stagg, C.J. (2017). Driving human motor cortical oscillations leads to behaviorally relevant changes in local $GABA_A$ inhibition: a tACS-TMS study. J. Neurosci. *37*, 4481–4492.

STAR★METHODS

KEY RESOURCES TABLE

REAGENT or RESOURCE	SOURCE	IDENTIFIER
Software and Algorithms		
MATLAB 2016a	The MathWorks, Natick, MA, USA	https://uk.mathworks.com/products/new_products/release2016a.html
SPSS 20	IBM Corporation, Armonk, NY, USA	https://www.ibm.com/analytics/us/en/technology/spss/
FMRIB Software Library (FSL) 5.0	Oxford Centre for FMRI of the Brain (FMRIB), Wellcome Centre for Integrative Neuroimaging (WIN), Oxford, UK	https://fsl.fmrib.ox.ac.uk/fsl/fslwiki

CONTACT FOR REAGENT AND RESOURCE SHARING

Further information and requests for resources should be directed to and will be fulfilled by the Lead Contact, Dr Charlotte Stagg (charlotte.stagg@ndcn.ox.ac.uk).

EXPERIMENTAL MODEL AND SUBJECT DETAILS

12 healthy volunteers (7 male; mean age: 26 (range 21-42)) gave their informed consent to participated in the study in accordance with local ethics committee approval (NRES Committee South Central - Berkshire B; 13/SC/0413). Subjects were right-handed [35], had no previous neurological or psychiatric history and no contraindications to transcranial stimulation. Volunteers participated in two MRI sessions. The first session comprised three resting state scans, each lasting approximately 14 min. During each scan, four tACS periods of the same condition, each lasting 80 s (including 10 s ramp-up and ramp-down) were applied, with 110 s tACS-free periods between them (Figure 1). Current was switched off between the ramps for sham stimulation. The order of scans was counter-balanced across subjects. Subjects were asked to keep their eyes open and fixate on a cross presented in the center of the screen. Alertness, and possible tACS side-effects were assessed throughout the session (see Figure S1).

In order to identify functional ROIs, a second MRI session was performed.

METHOD DETAILS

MR acquisition

Whole-brain functional resting-state MRI (rs-fMRI) was performed using a multi-band echo planar imaging (MB-EPI) sequence on a 3T MRI system (Magnetom Verio 3T, Siemens) using a 32-channel head coil. For registration purposes a high-resolution T1-weighted 3D structural image was also acquired.

Resting state imaging

Whole-brain functional resting-state MRI (rs-fMRI) was performed using a multi-band echo planar imaging (MB-EPI) sequence (TR: 1300 ms, TE: 40 ms, 72 × 2 mm axial slices providing whole-brain coverage, FOV: 212 mm x 212 mm, voxel size: 2x2x2 mm) [36, 37]. During acquisition, subjects were instructed to lie awake and still in the scanner while fixating on a central fixation cross. For registration purposes a high-resolution T1-weighted 3D structural image (TR: 2040 ms, TE: 4.7 ms, flip angle: 8°, field of view: 192 mm x 192 mm, voxel size: 1x1x1 mm) was also acquired for every participant.

Functional Localizer scan

This session consisted of a sequential externally guided unimanual finger tapping task. Subjects were requested to tap either with their right or left index finger on a button box at 1 Hz. The speed of the tapping was indicated via a green blinking dot. The session consisted of 6 tapping blocks interspersed with 6 rest blocks, each lasting 30 s (total scanning time approximately 6 min). Before each tapping block subjects were instructed to start tapping with either the right or left hand via a short instruction displayed on the screen.

Transcranial alternating current stimulation (tACS)

An MRI-compatible DC-Stimulator-plus (Neuroconn GmbH, Germany) was used to deliver a 1 mA current via two 5x7 cm conductive rubber electrodes. One electrode was placed over the left primary motor cortex [M1; C3 position according to the International 10-20 EEG system]; the other electrode was fixed over the right supraorbital ridge. We used a sinusoidal waveform without DC offset, resulting in a mean current density (peak-to-peak) of 0.029 mA/cm^2 under the electrodes. While we did not continuously record impedance throughout the study, it was kept between 10 and 20 kΩ at all times, in line with local operating practice.

In order to assess the subjects' alertness during the scans, the question ‘‘Are you tired’’? was displayed on the screen for 5 s, 10 s before each stimulation block. Subjects were asked to respond via a hand-held button box using the index finger (‘‘yes’’) or middle finger (‘‘no’’) of their right hand in order to give a response. After each scan participants were asked to rate their experience of paraesthesia, discomfort, and the strength of phosphenes from 0 – 10 on visual analog scales with anchors (0: ‘‘no paraesthesia,’’ ‘‘no pain,’’ ‘‘no phosphenes’’; 10: ‘‘high paraesthesia,’’ ‘‘high pain,’’ ‘‘strong phosphenes’’; see Figure S1).

Image analysis

All data were analyzed using FSL tools (http://www.fmrib.ox.ac.uk/fsl) [38, 39]. Individual preprocessing steps included motion correction using MCFLIRT [40], non-brain tissue removal using BET [41], non-linear high-pass temporal filtering equivalent to 150 s (0.007 Hz) and spatial smoothing using a Gaussian kernel of FWHM 5.0mm. FMRI volumes were registered to T1-weighted high-resolution individual subject anatomical images using linear registration, and then to standard MNI152 template image using FMRIB's Nonlinear Image Registration Tool (FNIRT) [39].

Next, denoising was performed using Independent Component Analysis (ICA), as implemented in FSL. Independent Component Analysis (ICA), as implemented in FSL was run for each subject and each scan separately. Each component from the ICA was classified as either signal or noise in two ways to ensure reproducibility. First, identification of artifactual components was performed manually by a blinded researcher. However, the decision of whether a component is classified as signal or as noise requires a high level of expertise of signal and noise fluctuations' spatiotemporal characteristics, and can therefore be confounded by subjective judgment, several ‘‘full-automatic’’ approaches to ICA classification have been developed [42]. To ensure the reliability of our ICA classification we applied FIX (FMRIB's ICA-based Xnoiseifier) [43]. A comparison between the automatic and manual ICA classification revealed an inter-rater co-efficient of 0.95, demonstrating the accuracy of our manual ICA classification. The artifactual components identified from our manual approach were then removed. As the model-free ICA approach does not allow the inclusion of regressors of no interest for the visual display and the subsequent button press response we excluded the volumes where these events occurred [44].

Analysis of brain activity changes

Voxel-wise analyses. Region of Interest (ROI) masks were defined as follows:

Primary motor cortex (M1): We determined the group mean activity within the left M1 during tapping of the right finger, which was then masked by a generous anatomical mask of the left M1 [45]. We then extracted the peak voxel and created a 10x10x10 mm ROI centered on these co-ordinates. The mask for the right M1 was defined similarly from the left tapping data.

Premotor cortex (PMC): We derived PMC masks from a connectivity-based parcellation of the premotor cortex [46], mapped onto the standard space MNI template.

To investigate changes in brain activity induced by tACS we used a General Linear Model (as implemented via FEAT in FSL [38]). The first level model consisted of one boxcar regressor modeling the tACS stimulation.

A second level mixed-effects analysis was used to calculate group mean maps of areas changed activity during stimulation versus baseline across all subjects for each stimulation condition separately. In addition, a second level mixed-effects analysis was performed to investigate differences in activation between stimulation conditions.

Region of interest (ROI) analyses. Given our strong a priori hypothesis as to the anatomical distribution of tACS-induced brain activity changes based on the known connectivity of M1, we also performed a ROI analysis to investigate potential effects of tACS on brain activity in the motor network.

The mean percentage BOLD signal change across all activated voxels within each ROI was calculated for each subject. BOLD changes were then compared between conditions using a repeated-measures ANOVA with one factor of tACS frequency (20 Hz, 5 Hz or sham) and one factor of ROI (left M1, right M1, left PMd and right PMd). When sphericity was violated appropriate corrections were performed.

Functional Connectivity analysis

Group ICA. In order to investigate whether tACS induced connectivity changes within brain networks the concatenated fMRI dataset was analyzed using ICA as implemented in MELODIC [17]. Data were decomposed into 20 components and RSNs of interest were identified using spatial correlations against previously defined maps [17]. A dual-regression approach was used to identify subject-specific RSN maps for each tACS condition [47, 48].

The subject-specific motor network and DMN RSN was then masked by the corresponding group mean RSN and the mean value within this region extracted for each subject, giving a measure of the strength of functional connectivity within each of the selected RSNs [21].

To assess network strength of the non-primary motor regions, the subject-specific motor network was masked first by both the group mean RSN as an inclusion mask, and then by an anatomical M1 mask as an exclusion mask [45]. The mean strength within the remaining non-primary motor regions was then calculated as above.

Seed-based correlations. Because we were primarily interested in connectivity changes between the stimulated M1 and other key nodes within the motor network we investigated connectivity changes between the left M1 and right M1 and between the left M1 and left PMd. We extracted the individual time course of the fMRI signal from the ROIs as described above. A Pearson's correlation

analysis was performed on the time-courses from the left and right M1; and the left M1 and the left PMd. As this is non-normally distributed we performed a Fisher's r-to-Z transformation prior to running statistical analyses.

Phase-precession model

There is a growing body of evidence that implicates phase-precession as a physiologically plausible mechanism for the emergence of slow-frequency fluctuations in MEG and BOLD fMRI [33, 34]. These dynamics have been modeled using Kuramoto-style oscillators [49] that have faithfully reproduced amplitude fluctuations as emerging from metastable oscillatory dynamics. In order to demonstrate that our hypothesis is compatible with such a phase-precession framework, we employed a Kuramoto-style model with dynamics given by,

$$\frac{d\theta_i}{dt} = \omega_i + \sum_j K_{ij} \cdot \sin(\theta_j - \theta_i) + \epsilon_i$$

where θ_i represents the phase of the ith oscillator, ω_i is the natural frequency of that oscillator, K_{ij} is the strength of influence of node *j* on node *i*, and ϵ_i represents a $\mathcal{N}(0, \sigma)$ noise source. Our network incorporated five oscillator nodes, whose topography is shown in Figure 4A. All nodes received independent noise of standard deviation σ, excepting the tACS node where $\sigma_{tACS} = 0$.

Model parameters were selected to simultaneously 1) maximize the correlation between M1-M1 and the broader motor network in the absence of stimulation, 2) minimize the correlation between M1-M1 and the broader motor network during 20 Hz stimulation, 3) reduce SM↔SM coupling during 20 Hz stimulation and 4) minimize the change in M1-M1 coupling due to 20 Hz stimulation. We are interested in the coupling between nodes reflected in their relative phase-alignments, which will be adopted as a reasonable surrogate of the low-frequency covariation in BOLD within this simulation. This is based on the premise that phase synchronization across neural populations (or coupled oscillators as here) leads to increased post-synaptic efficacy through temporal integration and thereby improved communication, so that low frequency dynamics in phase synchronization should be translated to low frequency amplitude covariations detectable in the BOLD signal [49]. To be explicit, BOLD was not used here to track beta oscillations *per se.* Rather we used it to follow emergent dynamics in neural activity at low frequencies arising through intrinsic modulations in the phase synchronization at higher frequencies, or due to small frequency mismatches between intrinsic beta activities and the 20 Hz drive provided by tACS. Both lead to low frequency modulations in the envelope of phase synchronization. In the case of tACS this modulation will have a frequency that is related to the mismatch between the frequency of intrinsic beta activities and the beta tACS. The stimulation frequency at 20 Hz was carefully chosen so that it was very close to the peak frequency of beta (mean 20.1 Hz, SD = 2.07, n = 20) recorded over M1 in healthy control subjects of similar age in our lab [50], thereby constraining phase drifts to the frequency band that can be detected in the BOLD signal.

The objective of this simulation was to demonstrate, in line with our hypothesis, that the phase-precession model is capable of reproducing the behavioral phenomena observed in this study. Thus, although the model was optimized as a deterministic system (using a fixed random seed), the model nevertheless illustrates that topologies exist in a phase-precession framework that are compatible with our experimental observations and hypotheses. In addition, different random seeds were selected to generate the realizations depicted in Figure 4, thus the model appears broadly generalizable. Parameters include the natural frequencies ω_i (allowable range 18.0-22.0 Hz; except tACS which was fixed at 20.0 Hz), connection weights M1-M1 (symmetric), M1→SM, SM→M1, tACS→M1, and the standard deviation of noise σ. Connection weights were constrained between [-1, 0] which facilitated attractor dynamics. Optimization was conducted in two passes first through a custom written swarm optimization routine, then an interior-point algorithm. Once optimized, 100 sample simulations of 10 s were generated under fixed initial conditions but with 10 s burn-in. We quantified the net change in phase synchronization index (PSI) due to stimulation versus no stimulation using Cohen's d, correlations by Pearson's Product-Moment method, and differences in correlations due to stimulation by normalizing Pearson's correlation coefficients by Fisher's z-transform and computing the z-score of differences with associated two-tailed statistics. The model was constructed, simulated and analyzed using MATLAB (R2016a, Mathworks, USA).

The derived parameters of the model were – Frequencies: 19.7 Hz (M1(L)), 18.9 Hz (M1(R)), 20.5 Hz (SM), 21.3 Hz (SM); Connection strengths: -0.289 (M1(L) ↔ M1(R)), -0.473 (M1 → SM), -0.604 (SM → M1) and -0.500 (tACS → M1(L)); Noise contribution, $\sigma = 0.395$.

QUANTIFICATION AND STATISTICAL ANALYSIS

Whole Brain Analyses

For all group-level analyses, Z-statistic images were thresholded using clusters determined by Z > 2.3 and a (corrected) cluster significance threshold of p = 0.05 within FSL.

Region of Interest Analyses

Statistical analyses were performed using SPSS (version 20.0) and the statistics toolbox of MATLAB (version 7.11.0.584). Connectivity changes were compared between conditions using repeated-measures ANOVA with one factor of tACS frequency (20 Hz, 5 Hz or sham) and one factor of ROI (left M1, right M1, left PMd and right PMd).

Current Biology, Volume *27*

Supplemental Information

Modulation of Long-Range Connectivity Patterns

via Frequency-Specific Stimulation of Human Cortex

Christiane A. Weinrich, John-Stuart Brittain, Magdalena Nowak, Reza Salimi-Khorshidi, Peter Brown, and Charlotte J. Stagg

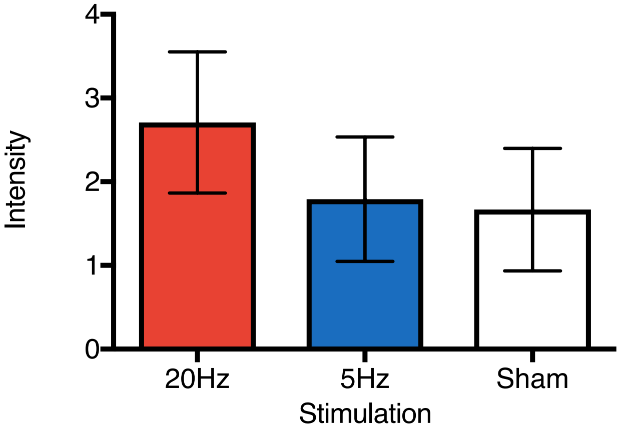
4
3
2
1
0
Intensity
20Hz
5Hz
Sham
Stimulation

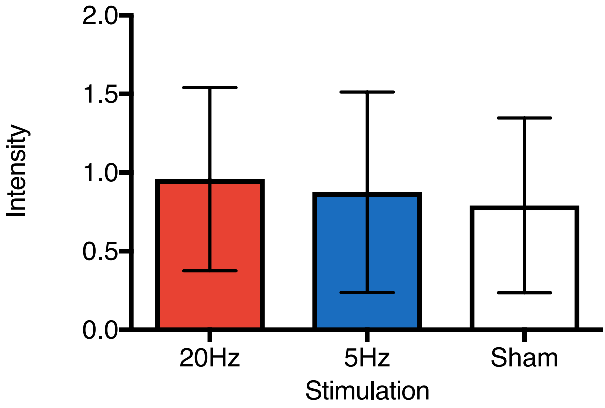
2.0
1.5
1.0
0.5
0.0
Intensity
20Hz
5Hz
Sham
Stimulation

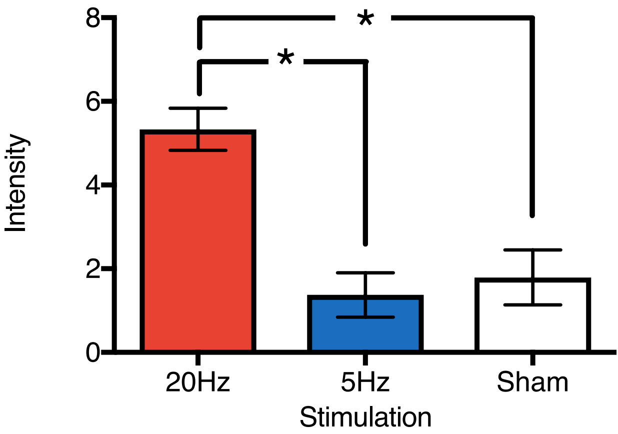
8
6
4
2
0
Intensity
*
*
20Hz
5Hz
Sham
Stimulation

Figure S1. Subject reported experience of tACS-induced phenomena, Related to Figure 1.

Paraesthesia (A), pain (B), and phosphenes (C) were rated on a visual analogue scale from 0 -10. There was no difference in the perception of paraesthesia and pain between the three stimulation conditions (20Hz, 5Hz and Sham) (Repeated Measures ANOVA: sensation: $F(2,22) = 1.73$, $p = 0.20$; pain: $F(1.108,12.186) = 0.33$ $p = 0.60$). However, there was a significant difference in the strength of phosphenes perceived between the three stimulation conditions (Repeated Measures ANOVA: $F(1.37, 15.01) = 28.52$, $p < 0.001$). Phosphenes perceived during tACS with this electrode montage are likely to be due to direct stimulation of the retina, rather than reflecting stimulation of the occipital cortex [S1]. Subsequent post hoc t-tests revealed 20Hz stimulation elicited a significantly stronger perception of phosphenes compared with both sham and 5Hz stimulation (20Hz versus sham: $t(11)=5.11$, $p < 0{,}001$; 20Hz versus 5Hz:, $t(11) = 6.20$, $p < 0.001$ [corrected]). There was no significant relationship between phosphene rating during 20Hz tACS and the strength of the motor network in response to 20Hz tACS ($r=0.32$, $p=0.30$), nor between phosphene rating and the strength of the DMN during 20Hz tACS (0.47, $p=0.11$).

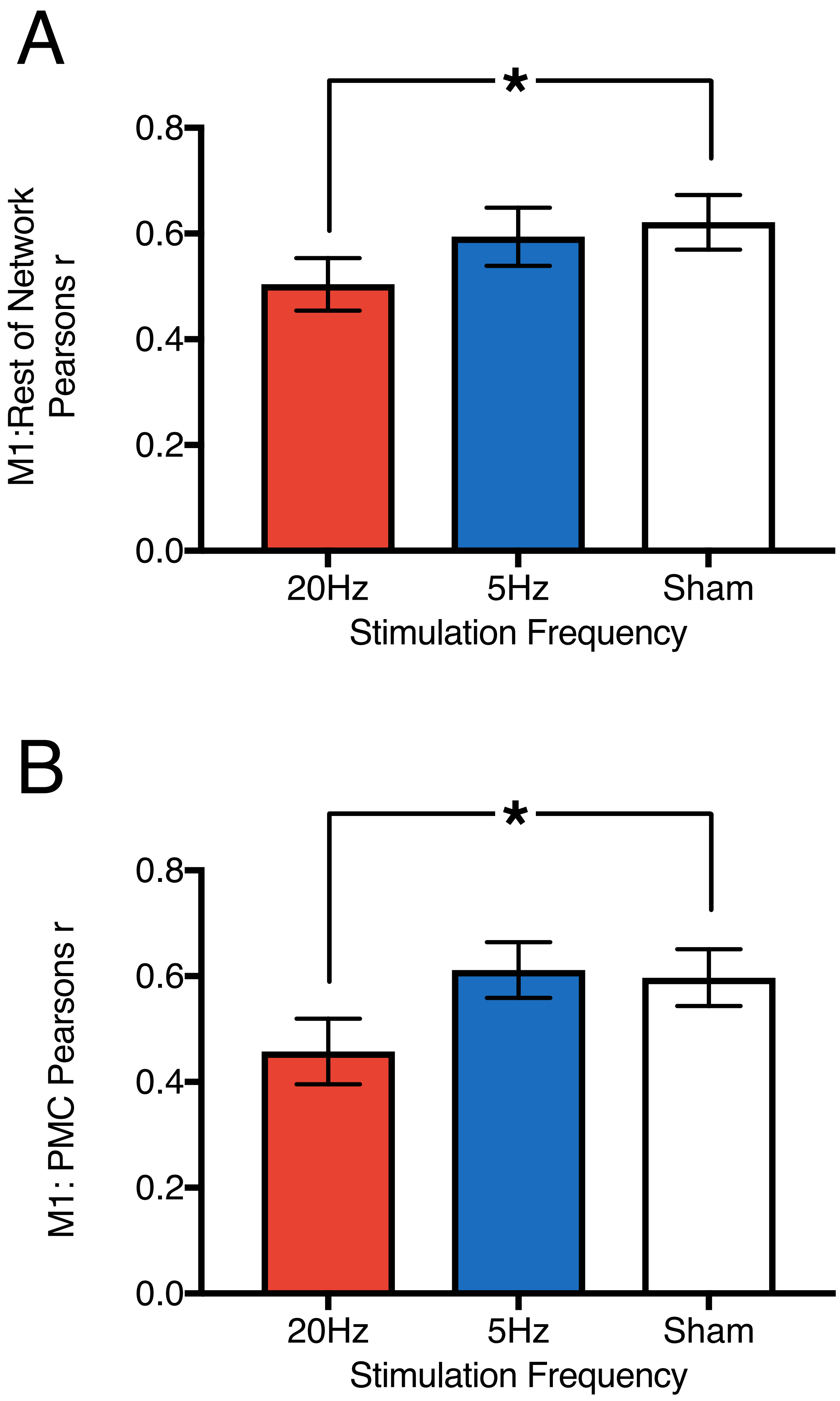
A
*
0.8
0.6
0.4
0.2
0.0
M1:Rest of Network
Pearsons r
20Hz
5Hz
Sham
Stimulation Frequency
B
*
0.8
0.6
0.4
0.2
0.0
M1: PMC Pearsons r
20Hz
5Hz
Sham
Stimulation Frequency

Figure S2. Region of Interest analyses. Related to Figure 2.

ROI analyses highlighting the change in connectivity between left M1 and (A) the motor network excluding the M1s bilaterally and (B) the left Premotor Cortex. Mean $\pm$ SEM, * $p < 0.05$.

Supplemental References

[S1] Schutter DJL, Hortensius R. (2010) Retinal origin of phosphenes to transcranial alternating current stimulation. Clin Neurophysiol 121, 1080–1084.
